# Neural Circuitry Underlying Waking Up to Hypercapnia

**DOI:** 10.3389/fnins.2019.00401

**Published:** 2019-04-26

**Authors:** Satvinder Kaur, Clifford B. Saper

**Affiliations:** Department of Neurology, Program in Neuroscience, Beth Israel Deaconess Medical Center and Harvard Medical School, Boston, MA, United States

**Keywords:** obstructive sleep apnea, arousal, parabrachial nucleus, calcitonin gene related peptide, hypercapnia

## Abstract

Obstructive sleep apnea is a sleep and breathing disorder, in which, patients suffer from cycles of atonia of airway dilator muscles during sleep, resulting in airway collapse, followed by brief arousals that help re-establish the airway patency. These repetitive arousals which can occur hundreds of times during the course of a night are the cause of the sleep-disruption, which in turn causes cognitive impairment as well as cardiovascular and metabolic morbidities. To prevent this potential outcome, it is important to target preventing the arousal from sleep while preserving or augmenting the increase in respiratory drive that reinitiates breathing, but will require understanding of the neural circuits that regulate the cortical and respiratory responses to apnea. The parabrachial nucleus (PB) is located in rostral pons. It receives chemosensory information from medullary nuclei that sense increase in CO2 (hypercapnia), decrease in O2 (hypoxia) and mechanosensory inputs from airway negative pressure during apneas. The PB area also exerts powerful control over cortical arousal and respiration, and therefore, is an excellent candidate for mediating the EEG arousal and restoration of the airway during sleep apneas. Using various genetic tools, we dissected the neuronal sub-types responsible for relaying the stimulus for cortical arousal to forebrain arousal circuits. The present review will focus on the circuitries that regulate waking-up from sleep in response to hypercapnia.

## Introduction

Obstructive sleep apnea (OSA) is caused by a sleep state-dependent reduction in the pharyngeal dilator muscle activity that leads to the closure of the upper airway in the susceptible individuals. These recurrent episodes of complete or partial obstruction of the upper airway lead to the airway collapse, which causes periodic hypoxia and hypercapnia during sleep, causing brief arousals that restore airway patency (Schulz, 2010; Mannarino et al., 2012; White and Younes, 2012; White, 2017; Darquenne et al., 2018; Pham et al., 2018). These repeated arousals result in sleep disruption, which in turn causes cognitive impairment as well as cardiovascular and metabolic morbidities (Bonnet, 1985; Fletcher, 1996; Bennett et al., 1998; Malhotra and White, 2002; Jun and Polotsky, 2009; Malhotra and Loscalzo, 2009; Drager et al., 2010; Bonsignore et al., 2012). The transient cortical arousals and sleep fragmentation are associated with the autonomic dysregulation, increased oxidative stress and hemodynamic changes during sleep. In patients with OSA, these consequences have been linked to increased daytime sleepiness, cardiovascular and metabolic morbidities. Due to this, many OSA patients are also at risk of developing arterial hypertension, coronary heart disease, stroke, type 2-diabetes and mortality.

Although OSA can be treated effectively with continuous positive airway pressure, many patients do not tolerate it and compliance is often poor. One alternative therapeutic approach in OSA may involve modifying the arousal threshold that may augment respiratory drive during apnea and recruiting the upper air way muscles to reestablish stable breathing (Horner et al., 1991; Loredo et al., 1999; Horner et al., 2017; Sands et al., 2018; Zinchuk et al., 2018). However, designing drugs that can selectively reduce cortical arousals while maintaining or augmenting the respiratory drive during these respiratory events would require understanding the circuits that mediate cortical EEG and respiratory responses to apnea. This review will focus on recent attempts to identify that circuitry, in particular, using newer methods such as optogenetics and chemogenetics that allow selective, genetically directed targeting of the neuronal nodes that mediate cortical EEG and respiratory responses to apnea.

**FIGURE 1:**
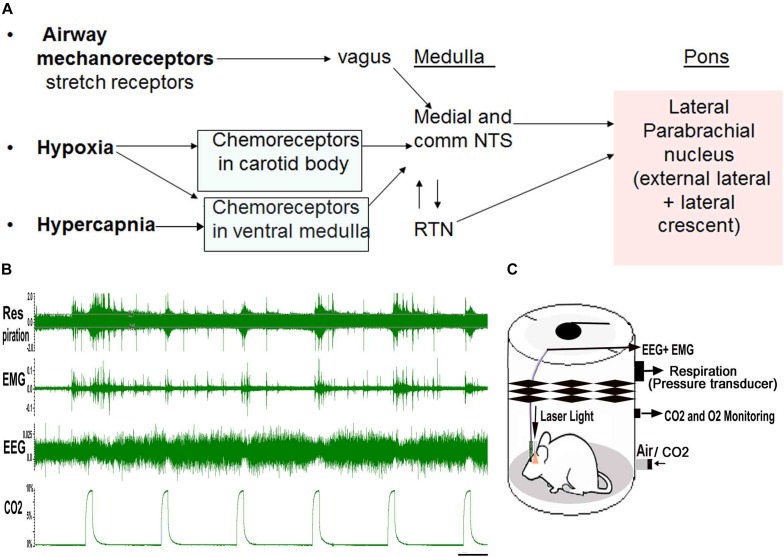
**(A)**, Three main stimuli related to apnea converge on the parabrachial area: Increased CO_2_ (Hypercapnia), Hypoxia and negative air-way pressure cause activation of both central and peripheral chemoreceptors whose signals are integrated in the nucleus of the solitary tract (NTS) and retrotrapezoid nucleus (RTN). The NTS and RTN activate neurons in the lateral parabrachial nucleus, major node in the brain stem that relay visceral sensory information to the forebrain areas. Mouse model of apnea: **(B)**, shows the “repetitive CO2 arousal (RCA) protocol” where a mouse is recorded in the plethysmograph chamber for the EEG, EMG and breathing, while exposed to repeated bouts of CO2 (hypercapnia). Mice undergo spontaneous periods of sleep and wake, however, only trials where the mouse is in NREM sleep for at least 30 s prior to onset of the CO2 are used to examine arousal. During these trials, the arousals are judged by EEG arousal (loss of delta waves and appearance of low voltage fast EEG), which is usually accompanied by EMG activation. Scale = 45 s **(C)**, is a schematic of the plethysmography chamber used to model apnea in mice, while they are exposed to CO2 and recorded for EEG/EMG and breathing responses, with and without laser light that is transmitted through the pre-implanted optical glass fibers. [Adapted and modified from Kaur et al. (2013)]. Kaur et al. (2013), is published under Creative Commons Attribution-Non-commercial-Share License, and therefore no permission is required reproducing this modified version.

The brain circuitry that underlies waking up to hypercapnia (increased CO2) that can occur in apnea is not clearly understood. Briefly, three main sensory stimuli that alert the brain during apnea are hypoxia, hypercapnia and negative air pressure in the airways created due to increased respiratory efforts (sensed by mechanoreceptor fibers in vagus) during apneas (White, 2006, 2017). The carotid body primarily senses the hypoxia and to a lesser extent the hypercapnia, and transmits that information to the nucleus of the solitary tract (NTS) via the carotid sinus branch of the glossopharyngeal nerve (Massari et al., 1996; Lindsey et al., 2018). In addition, the chemosensory neurons in the retrotrapezoid nucleus (RTN) directly sense the CO2, and these project in parallel to the NTS to the ventrolateral medulla (VLM- pattern generator for breathing), and parabrachial nucleus (PB- relay node for visceral sensory information from the brainstem to the forebrain) (Dean et al., 1989; Herbert et al., 1990; Finley and Katz, 1992; Massari et al., 1996; Guyenet et al., 2010a; Bochorishvili et al., 2012; Guyenet and Bayliss, 2015; Lindsey et al., 2018; Figure 1A). The serotonergic raphe system in the brainstem (Richerson et al., 2001; Depuy et al., 2011) and orexin neurons in the lateral hypothalamus (Hunt et al., 2016; Rodrigues et al., 2019), are other CO2 sensing neurons, which also project to the NTS, the VLM, and the PB. In patients with OSA, arousal correlates closely with the airway negative pressure, to a lesser degree to the level of hypercapnia, and least with the level of hypoxia (Gleeson et al., 1990). However, all the three stimuli converge in the same brain locations; therefore studying these areas and their connections is important to understand the brain response to apnea.

## Parabrachial Nucleus and Cortical Arousal

The PB, a relay node for sensory visceral information, that surrounds the superior cerebellar peduncle, is referred to as the “Pontine taste area” (Saper, 2016) and the same region as early as 1920s was also identified as the “pneumotaxic center” or the “pontine respiratory group” by the workers on respiratory control (Feldman et al., 1976; Dobbins and Feldman, 1994). Recent studies have associated the PB with cortical arousal. In addition to the canonical, cholinergic (Semba and Fibiger, 1992; Steriade et al., 1993; Kleiner and Bringmann, 1996; Datta and Siwek, 1997; Cape and Jones, 2000) and monoaminergic (Aston-Jones and Bloom, 1981; Berridge and Wifler, 2000) arousal pathways from the upper brainstem, the PB projects to the thalamus, hypothalamus, and cerebral cortex (Saper and Loewy, 1980; Steriade et al., 1993; Jones, 2005). Surprisingly, cell specific lesions of the cholinergic and monoaminergic neurons, either alone or in combination, in these pontine areas have been found to cause little alteration in wake in both cats and rats (Jones et al., 1973; Holmes and Jones, 1994; Lu et al., 2006; Blanco-Centurion et al., 2007; Fuller et al., 2007), whereas large PB lesions cause profound coma (Fuller et al., 2011). Studies from our lab and others have shown that cortical arousal can be induced by activation of PB (Hayashi et al., 2015; Qiu et al., 2016; Kaur et al., 2017) and the deletion of glutamatergic signaling in PB neurons increases sleep and causes EEG slowing (Fuller et al., 2011; Kaur et al., 2013). Therefore, ascending projections of the PB through a ventral forebrain pathway via the hypothalamus and BF may play a key role in mediating cortical arousal. As the PB consists of different diverse sub nuclei, each with its distinct input and output targets, and these are often associated with different neuromodulators (Fulwiler and Saper, 1984), it is therefore, necessary to further dissect it using newer genetically specified tools to understand the roles of different cell types in this functionally heterogeneous population.

## Parabrachial Nucleus and Breathing

PB receives chemosensory information from RTN and NTS, that sense hypercapnia and hypoxia and also from the upper airway afferents that respond to pulmonary negative pressure associated with apneas (Panneton and Loewy, 1980; Finley and Katz, 1992; Mizusawa et al., 1995; Berquin et al., 2000; Pete et al., 2002; Izumizaki et al., 2004; Rosin et al., 2006; Corcoran et al., 2009; Song and Poon, 2009; Gonzalez et al., 2010; Guyenet et al., 2010b; Topchiy et al., 2010; Bochorishvili et al., 2012; Yokota et al., 2012, 2015; Guyenet and Bayliss, 2015; Roman et al., 2016). As mentioned above, the PB area not only exerts powerful control over cortical arousal (Fuller et al., 2011; Kaur et al., 2013, 2017; Hayashi et al., 2015; Qiu et al., 2016) it also regulates respiration (Miura and Takayama, 1991; Chamberlin and Saper, 1994; Mizusawa et al., 1995; Chamberlin, 2004; Bonis et al., 2010a,b; Diaz-Casares et al., 2012; Damasceno et al., 2014; Kaur et al., 2017; Yang and Feldman, 2018). The ascending projections of the PB mediate cortical arousal (Saper and Loewy, 1980; Saper, 1982; Kaur et al., 2013, 2017; Saper, 2016), while its descending projections to the respiratory areas such as ventral lateral medulla, hypoglossal motor nucleus and phrenic motor nucleus, may regulate respiration (Yokota et al., 2001, 2012, 2015). Thus, the PB is an excellent candidate for a site that can augment the airway dilator muscles, particularly following EEG arousals during sleep apneas. However, the precise brain circuitries that can be selectively targeted to prevent cortical arousal but augment respiration and maintain air-way patency during apneas need to be investigated.

## Mouse Model of APNEA

We designed a mouse model of apnea (Kaur et al., 2013) to simulate breathing during apneas and understand the brain circuitry underlying the repetitive arousals during apnea. Briefly, a mouse is kept in a plethysmograph chamber, and every 300 s, the gas mixture entering the chamber is switched for 30 s to one that contains either increased CO2 (10%), reduced O2 (10%) or both. The gasses mix in the chamber and approach the new steady state after about 10–15 s. At the end of the 30 s period, the source is switched back to air, and the gas levels return to baseline. We continuously record the EEG and EMG, the plethysmograph (which gives us tidal volume and respiratory rate) and percentage of CO2 and O2 in the chamber (Figure 1). In our earlier study, the arousal kinetics to CO2 (hypercapnia alone) and to the combined hypercapnia and hypoxia were identical (Kaur et al., 2013), therefore we continue to test EEG arousals with hypercapnia in our model (Figure 1B,C), and we will refer here only to the trials with elevated CO2. We used this paradigm of repetitive hypercapnia as a model of sleep apnea as the duration of the gas disturbance, its frequency, and the length of the arousals, were similar to those seen in a patient with mild sleep apnea. Also hypercapnia is mechanistically more relevant than hypoxia in sleep disordered breathing related neuro-impairment (Wang et al., 2014, 2016), even though the effect of hypoxia only is more extensively studied by most groups.

## Genetic Tools and Technologies for Circuit Analysis

**FIGURE 2:**
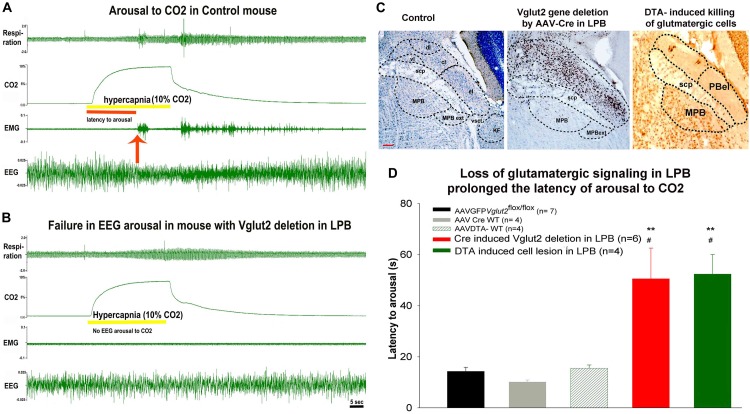
Testing the role of glutamatergic signaling in hypercapnia induced arousal: **(A,B)**, are the two representative trials from a control mouse **(A)**, where cortcial EEG arousal in response to hypercapnia occurs in 15 s after onset of CO2, while the mouse with deletion of Vglut2 gene in the LPB **(B)**, fails to wake wake up to hypercapnia. **(C)**, Photomicrograph of the Nissl-stained coronal section of the mouse brain, showing different sub divisions of the parabrachial (PB) nucleus, Cre-immunoreactivity (brown) against a Nissl-stained background (blue) in the neurons in the lateral parabrachial (LPB) region after injection of AAV-Cre in *Vglut2* ^flox/flox^ mice and last panel shows the shows a photomicrograph of a brain section immunostained for Neu-N, a neuronal marker after bilateral injection of AAV-DTA killed *Vglut2*+ neurons into the LPB. **(D)**, Show graphs of the latency of arousal during and after a hypercapnic stimulus of 30 s in mice injected bilaterally with AAV-DTA (green) compared to the control (black, gray, and striped green) and LPB group from which *Vglut2* was deleted in the LPB including the PBel (red). scp – superior cerebellar peduncle; dl – dorso-lateral; cl – centro-lateral; el – external lateral; vl – ventrolateral PB subnucleus; MPB – medial and MPB-ext – medial external-lateral parabrachial nucleus; KF – Kolliker Fuse; vsct – ventral cerebro-spinal tract; Scale = 100 μm. ^∗∗^represents *p* < 0.01 compared to the control group (AAV-GFP) and ^#^*p* < 0.05, compared to the AAVCreWT group. [Adapted and modified from Kaur et al. (2013)]. Kaur et al. (2013), is published under Creative Commons Attribution-Non-commercial-Share License, and therefore no permission is required reproducing this modified version.

In the past, researchers have used a wide range of electrophysiological and molecular tools, either individually or in combination, to probe and manipulate neural circuits. Although, these had helped us understand some basic pathways, the inherent heterogeneity of brain cells and the lack of target specificity of these earlier tools make the interpretation of such data difficult. For example, pharmacological approaches using receptor agonists and antagonists have been confounded by poor blood-brain permeability when given systemically; low solubility when given directly into the brain; and “off-target” side effects when they engage unintended targets. Other approaches that involve the use of the global gene knockout, sometimes suffer from low temporal and spatial resolution and such approaches can also be confounded by ontogenetic and ectopic expression of the gene of interest. Similarly, both acute and chronic lesions produce collateral damage to adjacent brain structures making it difficult to interpret the effects which could be secondary to the lesion itself. Now, the emergence of newer conditional genomic models and viral-vectors approaches allow us to precisely target a selective cell population in the brain area of interest, and this is helping to link specific group of neurons and neural pathways to specific behaviors (Carter et al., 2010, 2013b; Carr and Zachariou, 2014; Fuller et al., 2015; Han et al., 2015; Campos et al., 2016, 2018; Qiu et al., 2016; Whissell et al., 2016; Saper and Fuller, 2017; Wu et al., 2018). The introduction of the Cre transgenes through gene delivery methods using the Cre/ lox system provides better temporal and spatial control over Cre-mediated excision of a selective gene sequence encoding the protein of interest (Kaur et al., 2013; Abbott et al., 2014; Todd et al., 2018), in a selective brain area (Figure 2C).

In recent years, the use of chemo- and opto-genetic tools had equipped us with an unparalleled ability to probe the neural circuitry that underlies behavioral state. The genetically engineered receptors are successfully used as tools for targeting chemo- and opto-genetics to selective cell types. Because they are activated either by injectable synthetic ligands that specifically bind to these receptors on the targeted cells and excite or inhibit them (chemogenetics) or through the delivery of specific wavelengths of laser-light via an implanted optical fiber (optogenetics), investigators retain temporal control over particular subsets of neurons (Fuller et al., 2015; Park and Carmel, 2016; Vlasov et al., 2018). In addition, because the opto- or chemogenetic tool is expressed in a conditional manner, it is only expressed by cells of a specific genotype, thus giving the investigator both neuroanatomical and neurochemical control over the response. Because the receptor transcript is packaged within a Flip-Excision-Switch (FLEX) cassette, the functional receptor can be expressed only in the presence of cre-recombinase (Schnutgen et al., 2003; Fuller et al., 2015; Plummer et al., 2017). The use of the Cre-driver mouse lines, where cre is expressed downstream of a selected promoter, ensures that the designer receptors when injected in these mice are expressed in a Cre-dependent manner, specifically in neurons that express a select protein. Finally, because the viral vectors for the opto- and chemogenetic tools can be injected locally in the brain, the investigator also has spatial control over the part(s) of the brain involved in the experiment. These modified receptors can act as effective tools that allow us to manipulate a selective neural circuit and we can then evaluate the effect upon the behavior in direct relation to either the excitation or inhibition of a specific neuronal node.

## Glutamatergic Signaling in the PB and Waking Up to CO2

To examine the role of glutamatergic signaling in the PB, in one set of animals we deleted the vesicular glutamate transporter-2 (Vglut2) gene in various PB sub-nuclei (by injecting an AAV-Cre into the PB of Vglut2^flox/flox^ mice). In another set of mice, we killed the cells in the lateral PB using injections of AAV that had Cre-dependent expression of diphtheria toxin subunit A (DTA) in Vglut2-Cre mice (Figure 2C). We tested these mice for arousal to hypercapnia using the mouse model of apnea (Figure 1). Our results indicated that deletion of glutamatergic signaling from neurons in the external lateral PB (PBel), or killing the Vglut2 neurons in the PBel produced the same prolongation of the latency of waking up to CO2 (Figure 2A–D), suggesting that glutamate alone in PBel neurons is the operative neurotransmitter for relaying the signal for waking up from sleep in response to hypercapnia (Kaur et al., 2013). Many neurons in the PBel express calcitonin gene related peptide [CGRP, (Yasui et al., 1991); PBel^CGRP^], and we tested whether these are activated (cFos expression) in response to the hypercapnia (Yokota et al., 2015). Most of the cFos positive neurons in the PBel contained CGRP, while many along the lateral edge of the nucleus did not. Because most PBel^CGRP^ neurons project to the forebrain, whereas most neurons lateral to them project to the brainstem, we hypothesized that the PBel^CGRP^ neurons might be selectively responsible for forebrain arousal during hypercapnia.

## Role of the PBEL^CGRP^ Neurons in Cortical Arousal

**FIGURE 3:**
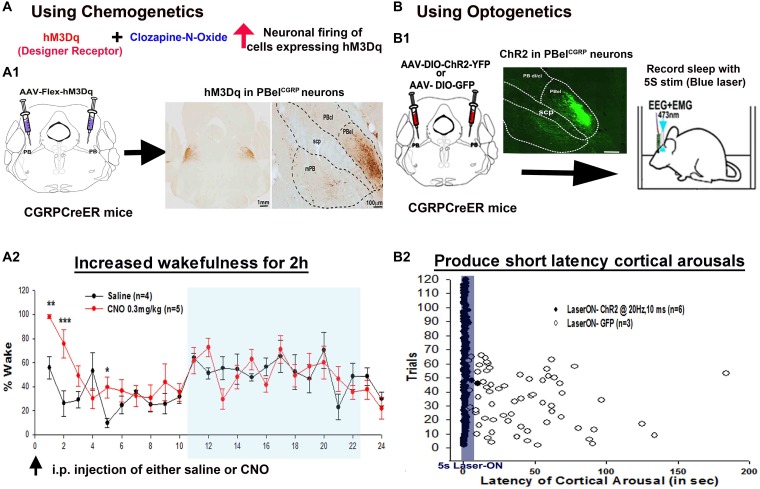
Selective activation of the PBel^CGRP^ neurons, using chemogenetics **(A)** and optogenetics **(B)**: The (**A1,B1)** represent the strategy used to first express either hM3Dq or Channel rhodopsin (ChR2) in the PBel^CGRP^ neurons, using the CGRP-CreER mice. Chemogenetic activation of PBel^CGRP^ neurons, significantly increased wakefulness for 2 h post injection of the designer ligand (CNO) that binds hM3Dq **(A2)**. Optogenetically driving the PBel^CGRP^ neurons also produced very short latency arousals both at 10 and 20 Hz, trials shown in the figure are from stimulation at 20 Hz **(B2)**. (^∗∗∗^*p* < 0.0001; ^∗∗^*p* < 0.001; 1-way or repeated measures ANOVA followed by Holm-Sidak for multiple comparison). [Adapted and modified from Kaur et al. (2017)]. Kaur et al. (2017) is published in a Cell Press journal “Neuron,” and no permissions needed to reproduce the modified versions of the published figure.

Using optogenetic and chemogenetic tools in CGRP-CreER mice (Kaur et al., 2017), we could selectively activate and inhibit the PBel^CGRP^ neurons. Optogenetic activation of PBel^CGRP^ neurons at 10 and 20 Hz by 10 ms blue laser light pulses (Figure 3B) caused short latency arousals and their chemogenetic activation significantly increased wakefulness (Figure 3A; Kaur et al., 2017). Targeting yellow (593 nm) laser light to the archaerhodposin –TP009 (ArchT) expressing PBel^CGRP^ neurons during the CO2 trials, silences them. Mice with inhibition of PBel^CGRP^ neurons (Laser-ON) failed to wake up to CO2 in 50% of the trials and increased the latency to arousal by four fold in response to the CO2 (Figre 4A,C). These results were similar to those we obtained with killing most of the lateral PB neurons, or deleting their Vglut2 gene. In other words, the PBel^CGRP^ neurons appear to provide most if not all of the arousal response to CO2, by using glutamate as their neurotransmitter.

**FIGURE 4:**
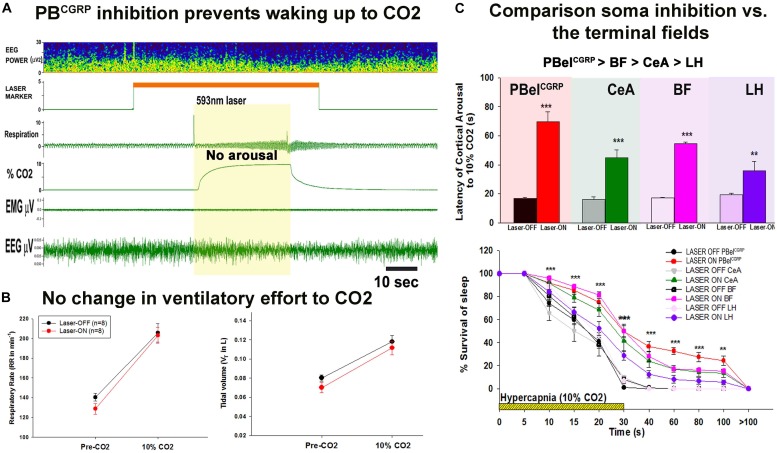
Selective silencing of the PBel^CGRP^ neurons and the terminal fields using Optogenetics: **(A)**, is the representative recording of EEG, EMG and respiration during the 10% CO2 stimulus in a CGRP-CreER mouse with laser photo-inhibition of PBel^CGRP^ neurons, the mouse in this representative trial did not wake up, and had similar increase in the ventilatory-drive in response to CO2 as the control with Laser-OFF. **(B)**, Left panel, are the graphs comparing the respiratory rate (RR) and the tidal volume (V_T_) (right panel) for 3 breaths before CO2 (Pre CO2) and for 3 breaths during CO2 just prior to waking-up in Laser-OFF and then at the same time point in trials in the same animal with Laser-ON (in which the animals did not awaken). **(C)**, Compares the effects of PBel^CGRP^ soma inhibition to that of PBel^CGRP^ terminals field inhibition **(A)**, latency of arousal mean (SEM) during laser (593 nm) induced inhibition of the PBelCGRP neurons is compared with inhibition of the terminal fields in the BF, CeA, and LH. **(B)** Survival of sleep curves during and after a hypercapnic stimulus shown with and without laser. (^∗∗∗^*p* < 0.0001; ^∗∗^*p* < 0.001; 1-way or repeated measures ANOVA followed by Holm-Sidak for multiple comparison). [Adapted and modified from Kaur et al. (2017)]. Kaur et al. (2017) is published in a Cell Press journal “Neuron,” and no permissions needed to reproduce the modified versions of the published figure.

Interestingly, the silencing of the PBel^CGRP^ neurons preserved the respiratory drive (Figure 4B) during the hypercapnia, with no differences in the tidal volume and respiratory rate (Kaur et al., 2017). Also these laser-induced inhibitions did not affect the arousal thresholds to acoustic stimuli or somatosensory and vestibular stimulation (Kaur et al., 2017). Recent work from Palmiter and colleagues suggests that the CGRP neurons may respond to pain and to other visceral stimuli (e.g., gastrointestinal upset or conditioned taste aversion) (Carter et al., 2015; Han et al., 2015; Campos et al., 2018) which has led to the suggestion that they may serve a more generalized central alarm function, waking up the brain when aversive visceral or noxious stimuli arise (Saper, 2016; Palmiter, 2018).

**FIGURE 5:**
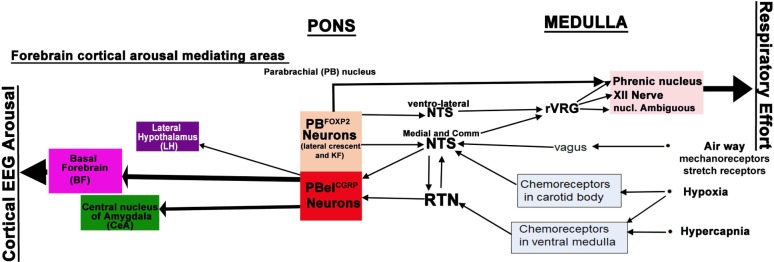
Neural circuitry regulating cortical arousals and respiratory efforts to hypercapnia: PBel^CGRP^ neurons receive CO2, O2, and airway mechanoreceptor inputs, as well as inputs via medullary nuclei, NTS (nucleus of solitary tract) and RTN (Retrotrapezoid nucleus). The PBel^CGRP^ neurons in turn project extensively to the lateral hypothalamus (LH), basal forebrain (BF), and central nucleus of amygdala (CeA). Based on our findings, PBel^CGRP^ neurons mainly cause cortical arousal by projections to the BF, which has potent waking effects, followed by CeA and LH. PBel^CGRP^ neurons did not contribute to respiratory component of apnea, as its inhibition did not diminish respiratory drive to CO2. The glutamatergic FoxP2 neurons (PB^FoxP2^) in the lateral crescent (PBlc) and Kolliker Fuse (KF) have descending projections to the hypoglossal nucleus (tongue), and to the retroambiguus (larynx) and phrenic motor nucleus (Diaphragm); they also project extensively to the ventro-lateral and commissural (Comm) subdivisions of NTS. These projections of the PB^FoxP2^ neurons may influence the respiratory efforts during apneas, either by direct projections to the motor neurons in medulla and spinal cord or by indirect projections to the premotor neurons in the ventro-lateral medulla, also known as the rostral Ventral Respiratory Group (rVRG).

To further investigate the arousal regulating circuitry targeted by PBel^CGRP^ neurons for causing arousals during apnea, we inhibited terminal fields at three major forebrain arousal nodes: the substantia innominata in the basal forebrain (BF); the central nucleus of the amygdala (CeA); and the lateral hypothalamus (LH). Optogenetic silencing of these terminals fields also increased the latency for arousal, with differential responses at multiple target sites. Our data suggested that PBel^CGRP^ neurons act most potently through their direct projections to the BF, whose neurons have direct projections to the cerebral cortex. The CeA also participates in the arousal, but has no ascending projections to the cortex or thalamus. Because it projects intensely to the BF, this is likely to be its mechanism of function. Lastly inhibition of the PBel^CGRP^ terminals in the LH field had the least effect on the arousal in response to CO2. Although some LH neurons directly project to cerebral cortex and others send axons to the BF, this region appears to play at most a minor role in arousal to CO2 (Figure 4C). Thus, the PBel^CGRP^ neurons are a critical node in the network that receives input from neural pathways activated in apneas in response to hypercapnia, hypoxia and airway stretching, and in relaying that influence to the forebrain sites to cause awakening during apneas (Figure 5).

PBel^CGRP^ neurons did not contribute to the respiratory component of apnea, as their inhibition did not diminish respiratory drive to CO2. Also, these neurons did not show any descending outputs to the respiratory areas, but adjacent neurons that showed a cFos response to CO2 in the lateral crescent (PBlc) and Kolliker Fuse (KF), do project to respiratory areas (Yokota et al., 2015). Of note, many of the neurons in this area express the transcription factor Forkhead-homeobox protein-2 (FoxP2), which is distributed throughout the respiratory column in both rats and mice (Geerling et al., 2017; Stanic et al., 2018; Figure 5). The glutamatergic FoxP2 neurons in the PBlc and KF have descending projections to respiratory areas such as the ventrolateral medulla including the pre-Bötzinger complex and retroambiguus area, the hypoglossal nucleus, the ventrolateral and commissural subdivisions of NTS, and the intermedio-lateral cell column (IML) and phrenic motor nucleus in the spinal cord (Geerling et al., 2017). These projections, much of which are likely to come from the PB^FoxP2^ neurons, may influence the respiratory efforts during apneas (Figure 5). However, it remains to be seen if selectively manipulating the PB^FoxP2^ neurons in the PBlc and KF can augment ventilatory efforts during hypercapnia. We are now investigating such a role of this population of PB^FoxP2^ neurons in augmenting respiration in response to hypercapnia and their possible interactions with PB^CGRP^ neurons.

Other brainstem cell groups, such as the serotonergic dorsal raphe (Richerson et al., 2005; Buchanan and Richerson, 2010; Ray et al., 2011, 2013; Smith et al., 2012) have also been shown to regulate hypercapnia induced arousals. A recent study showed that serotonergic dorsal raphe regulates waking up to CO2, and this is mediated through 5HT_2A_ receptors (Smith et al., 2018). However, mice deficient in 5HT neurons are responsive to the CO2 when injected with a 5HT_2A_ agonist (Buchanan et al., 2015), suggesting that DR serotonergic neurons are modulatory and maybe acting through a non-serotonergic area, e.g., PBel^CGRP^ neurons (Kaur et al., 2018). This possibility is also the subject of our current investigations.

## Conclusion

Effective pharmacotherapy for OSA will depend on identifying the sites that can selectively regulate the brain response to hypercapnia (Horner et al., 2017) and therefore be used as druggable targets. Another line of investigation, with the goal of providing more personalized therapeutic interventions for patients with a low arousal threshold (Sands et al., 2018), seeks to quantify and manipulate the “arousal threshold” in patients with OSA. The knowledge of selective neuro-circuitries that comprise functionally connected specific neurons, such as PBel^CGRP^ and PB^FoxP2^ neurons for regulation of cortical arousal and respiratory efforts during apnea can help with such interventions. Importantly, PBel^CGRP^ neurons are not only activated by hypercapnia, but are also responsive to various potentially dangerous or aversive stimuli (Carter et al., 2013b,a, 2015; Han et al., 2015; Campos et al., 2016, 2018; Saper, 2016; Palmiter, 2018). As such, it is plausible that different subpopulations of PBel^CGRP^ neurons encode and process different classes of aversive stimuli (Bernard et al., 1994; Campos et al., 2018) resulting in amplification of the hypercapnia-arousal response. Therefore, to treat a low arousal threshold in sleep apnea, there is a need for more precise understanding about the afferents that selectively modulate the PBel^CGRP^ neurons and therefore likely help tune the hypercapnia-arousal response. Thus, a deeper understanding of the PBel^CGRP^ neurocircuitry and it’s connections to other neuronal subpopulations in the PB (for e.g., PB^FoxP2^ neurons) and each of their distinct projection targets, will help yield valuable therapeutic targets, that can help prevent cortical arousal during apneas while preserving the respiratory drive important for restoring the airway patency. This will eventually help in preventing OSA and its negative secondary health consequences.

## Ethics Statement

All animal procedures met National Institutes of Health standards, as described in the Guide for the Care and Use of Laboratory Animals, and all protocols were approved by the Beth Israel Deaconess Medical Center Institutional Animal Care and Use Committee.

## Author Contributions

SK conceptualized, designed the experiments, collected and analyzed the data, and wrote the manuscript. CS contributed to the experimental concept and design, and wrote the manuscript.

## Conflict of Interest Statement

The authors declare that the research was conducted in the absence of any commercial or financial relationships that could be construed as a potential conflict of interest.
